# PPARα Protein Expression Was Increased by Four Weeks of Intermittent Hypoxic Training via AMPKα2-Dependent Manner in Mouse Skeletal Muscle

**DOI:** 10.1371/journal.pone.0122593

**Published:** 2015-04-29

**Authors:** Ge Li, Jianxiong Wang, Jianping Ye, Yimin Zhang, Ying Zhang

**Affiliations:** 1 Division of Exercise Biochemistry, Institute of Sports Science, Beijing Sport University, China; 2 School of Health and Wellbeing, Faculty of Health, Engineering, and Sciences, University of Southern Queensland, Australia; 3 Pennington Biomedical Research Center, Louisiana State University, Louisiana, United States of America; 4 Key Laboratory of Exercise and Physical Fitness (Beijing Sport University), Ministry of Education of the People’s Republic of China; East Tennessee State University, UNITED STATES

## Abstract

Peroxisome proliferator-activated receptor α (PPARα) is critical for muscle endurance due to its role in the regulation of fatty acid oxidation. The 5’-AMP-activated protein kinase (AMPK) is an energy sensor in cells, but its role in PPARα regulation *in vivo* remains unknown. In this study, we examined PPARα expression in the skeletal muscle of AMPKα2 overexpression (OE), knockout (KO) and wild-type (WT) mice after four weeks of exercise under intermittent hypoxia. WT, OE and KO mice were used at 40 mice/strain and randomly subdivided into four subgroups: control (C), running (R), hypoxia (H), and running plus hypoxia (R+H) at 10 mice/group. The treadmill running was performed at the speed of 12 m/min, 60 min/day with a slope of 0 degree for four weeks. The hypoxia treatment was performed in daytime with normobaric hypoxia (11.20% oxygen, 8 hours/day). In the R+H group, the treadmill running was conducted in the hypoxic condition. AMPKα2, phosphor-AMPKα (p-AMPKα) (Thr172), nuclear PPARα proteins were measured by Western blot and the medium chain acyl coenzyme A dehydrogenase (MCAD) mRNA, the key enzyme for fatty acid oxidation and one of the PPARα target genes, was also measured in skeletal muscles after the interventions. The results showed that nuclear PPARα protein was significantly increased by R+H in WT muscles, the increase was enhanced by 41% (p<0.01) in OE mice, but was reduced by 33% (p<0.01) in KO mice. The MCAD mRNA expression was increased after four weeks of R+H intervention. The change in MCAD mRNA followed a similar trend as that of PPARα protein in OE and KO mice. Our data suggest that the increase in nuclear PPARα protein by four-week exercise training under the intermittent hypoxia was dependent on AMPK activation.

## Introduction

Hypoxic training (combination of exercise and hypoxia) has been known to increase the endurance capacity of skeletal muscles in athletes [1,2]. Several studies have shown that hypoxic training improved the 5’-AMP-activated protein kinase (AMPK) and glucose transporter 4 (GLUT4) pathway to enhance glucose metabolism and mitochondrial function in the skeletal muscle of human or rats [3–5]. Considered of promoting aerobic metabolism of skeletal muscle and improving athlete performance, it is important to investigate the molecular mechanism by which hypoxic training affects skeletal muscle.

Peroxisome proliferator-activated receptor α (PPARα) is a ligand-activated transcription factor that belongs to the steroid hormone receptor superfamily [6]. PPARα is abundantly expressed in tissues with high fatty acid catabolism, such as liver, heart, and skeletal muscle [7], and is predominantly localized in the nucleus [8]. Over the last several decades, there have been many studies on the physiology, pharmacology, and functional genomics of PPARα. Both *in vivo* and *in vitro* studies have demonstrated that PPARα regulates the expression of some genes involved in lipid metabolism and plays a central role in the control of dyslipidemia associated with the metabolic syndrome [9–11].

PPARα may play an important role in exercise endurance. It has been reported that PPARα involved in the transcriptional regulation of metabolic enzymes and increased fatty acid oxidation in muscle tissues after endurance exercise training [12,13]. However, a down-regulation of PPARα protein was reported in skeletal muscle after a short-term (i.e. for a few hours to seven days) exercise in a normobaric hypoxic condition [14]. It remains unknown what happens to PPARα after long-term exercise in hypoxia, though the effects of long-term intermittent hypoxia alone on muscle performance has attracted much attention [15,16].

AMPK is an intracellular energy sensor that controls glucose and lipid metabolism, especially in skeletal muscles [17,18]. AMPK is a heterotrimeric kinase that contains two regulatory subunits (β and γ), and one catalytic subunit (α including α1 and α2). AMPK is activated in skeletal muscle during exercise to enhance ATP production through the phosphorylation of α subunit at Thr^172^ residue [19]. The role of PPARα in AMPK signaling pathway was investigated in a few studies in cellular models but the results of which were not conclusive. A study suggested that AMPKα activated PPARα in rat hepatocytes [20]. Another study reported that the activation of AMPK with 5-aminoimidazole-4-carboxamide ribonucleotide (AICAR), a well-established AMPK activator, increased PPARα activity in mouse skeletal muscle [21]. However, it was also reported that AMPK activation by AICAR reduced the transcriptional activity of PPARα in rat hepatoma cells [22]. In a review article, Leff cited unpublished data about inhibition of PPARα by AICAR [23]. These studies were all conducted *in vitro*, and the data have shown contradicting effects of AMPK on PPARα. To the best of our knowledge, there is no *in vivo* study to determine AMPK activation in the regulation of PPARα in skeletal muscle so far.

The purpose of the present study was to determine the role of AMPKα in the regulation of PPARα in skeletal muscle, through *in vivo* testing. PPARα protein and its target gene expression of medium chain acyl coenzyme A dehydrogenase (MCAD) mRNA (the key enzyme for fatty acid oxidation) were examined in AMPKα2 overexpression (OE) and AMPKα2 knockout (KO) mice (C57BL/6J) after four weeks of exercise training in normoxia or intermittent hypoxia. We tested the hypothesis of intermittent hypoxic training would achieve the stronger expressions of nuclear PPARα protein and its target gene compared to that of hypoxia or running only in mouse skeletal muscle; and the increases in all these expressions would be associated with AMPK activation.

## Materials and Methods

### Animal models

WT C57BL/6J mice and AMPKα2 OE mice were provided by the Institute of Laboratory Animal Science of Peking Union Medical College. The detailed method of the OE mice generation was described in our previous paper [24]. The AMPKα2 KO mice were kindly provided by Benoit Viollet (Department of Endocrinology, Metabolism and Cancer, Institute Cochin, University Paris Descartes, Paris, France) and bred by the Institute of Laboratory Animal Science of Peking Union Medical College. The generation of KO mice was described in detailed in the literature [25]. The AMPKα2 KO and AMPKα2 OE mice were both in C57BL/6J gene background. G*Power3 software (http://psycho.uniduesseldorf.de) was used to estimate the sample size. We chose power = 0.95 with α set at 0.05 and 12 groups, selected F tests, ANOVA: fixed effects, main effects and interactions, and then the result was computed to be seven mice per group with effect size = 0.6. In this study, there were 40 WT mice, 40 AMPKα2 OE mice, and 40 AMPKα2 KO mice. 10 mice were in each group.

All mice were two months old with a mean body weight of 18±2 g. They were housed with controlled room temperature (20–25°C), 12:12hr light—dark cycle and free access to food and water. The study protocol was approved by the Animal Care and Use Committee of Beijing Sport University, China.

### Hypoxic training protocol

After allowing acclimatization to their housing, each strain of the mice were randomly divided into four subgroups: control group (C), running group (R), hypoxia group (H), and running plus hypoxia (R+H) at 10 mice/group.

There was a 3-day adaptive training session before the interventions. During this session, the R and R+H groups run 15 minutes on a treadmill in day 1, 30 minutes in day 2, and 45 minutes in day 3, in normoxia or intermittent hypoxia, respectively. After the acclimation, the R and R+H groups were subject to 60 min/day treadmill running for four weeks at the speed of 12 m/min, with a slope of 0 degree. This exercise intensity corresponded to about 76% of maximal oxygen uptake in mice [26]. The hypoxia was made by placing the mice in a normobaric chamber (210 cm long, 200 cm wide, and 200 cm tall). The chamber was infused with 11.2% oxygen air through an air compressor (Ingersoll Rand, USA) which was connected with a hypoxic air machine. The oxygen concentration in the normobaric chamber was monitored throughout the experimental period with an oxygen sensor equipped with an alarm. The hypoxic treatment was 8 hours/day during daytime for four weeks. In the R+H group, mice completed the treadmill running in the normobaric hypoxic chamber. Before tissue collection, all mice had a 16-hour recovery time after the last intervention [24,27] with free access to food and water. The mice were euthanized by cervical dislocation and the quadriceps femoris muscles were collected, cleaned and quick-frozen in liquid nitrogen, and then stored at -80°C.

### Real-time PCR

Total RNA was isolated from about 50 mg of crushed muscle tissue using the TRI regent according to the manufacturer’s instructions. Real-time PCR was performed in a ABI 7500 Real-time PCR System (Life Technologies, USA) using the SYBR Green Real-time PCR Master Mix kit (Toyobo, Japan) with the previously synthesized cDNA (FSQ-101 Toyobo, Japan) as template in a 20μL reaction volume. Primers were used as follows: MCAD gene (QIAGEN, QT00111244) and 18s gene [28] (QIAGEN, QT010036875), qualified by software (ABI 7500 RT PCR). The difference in expression between control and experimental samples was calculated as 2^-ΔΔCT^, as did in a previous study of our laboratory [24]. To assess the specificity of the amplified PCR products, after the last cycle we performed a melting curve analysis and subjected reaction end products to electrophoresis in 1% agarose gels and compared band intensities by imaging of SYBR Green-stained gels.

### Western blot analysis

Total proteins were isolated from 50 mg of muscle using T-PER tissue protein extraction reagents (78510, Pierce, Rockford, IL, USA). Nuclear proteins were isolated from 50 mg of muscle using NE-PER nuclear extraction reagents (87792, Pierce, Rockford, IL, USA). Protein concentration was measured using the BCA protein assay kit (23225, Pierce, Rockford, IL, USA). After separated on a 10% SDS-PAGE by electrophoresis at 120 volts for 90 min, the fractionated proteins were transferred to a nitrocellulose transfer membrane. The membrane was blocked for 60 min in TBST (Tris-buffered saline with 0.10% Tween 20) containing 5% nonfat milk (5% Bovine Serum Albumin) to block AMPKα1/α2-Thr^172^ phosphorylation. The sample was then incubated overnight at 4°C using the following primary antibodies: anti-AMPKα2 (sc-19129, Santa Cruz Biotechnology, Santa Cruz, CA,USA), anti-phospho-AMPKα1/α2 (Thr172) (sc-33524, Santa Cruz, CA, USA), anti-PPARα (sc-9000, Santa Cruz, CA, USA), anti-β-actin (sc-47778, Santa Cruz, CA, USA), and anti- Histone1 (sc-10806, Santa Cruz, CA, USA). The membranes were then incubated with the appropriate anti-rabbit secondary antibody, diluted in TBST. Immunoreactive bands were highlighted by electrochemiluminescence (ECL) technology, exposed to light sensitive film for 15–90 s, and quantified by densitometry using image analysis software (Kodak Digital Science, New York, USA). The individual values were originally expressed as a ratio of a target protein and an internal protein standard (target protein content/internal protein content) and then expressed as a fold change of the normal oxygen WT control group (target protein content/internal protein content) value.

### Statistical analysis

Statistical calculations were performed using SPSS 13 (SPSS Inc., Chicago, IL, USA). A two-way ANOVA (strain x intervention) was used to analyse all of the variables across the experimental groups. The strain has three types: WT, OE, and KO. The intervention has four groups: C, R, H, and R+H. When a significant interaction effect was obtained, the simple main effect analysis with post-hoc (LSD test) was performed to identify significant mean differences between groups. A *p* value of 0.05 was considered statistically significant. The results were expressed as the means ± SD.

## Results

### AMPKα activation is induced by hypoxic training in skeletal muscle

**Fig 1:**
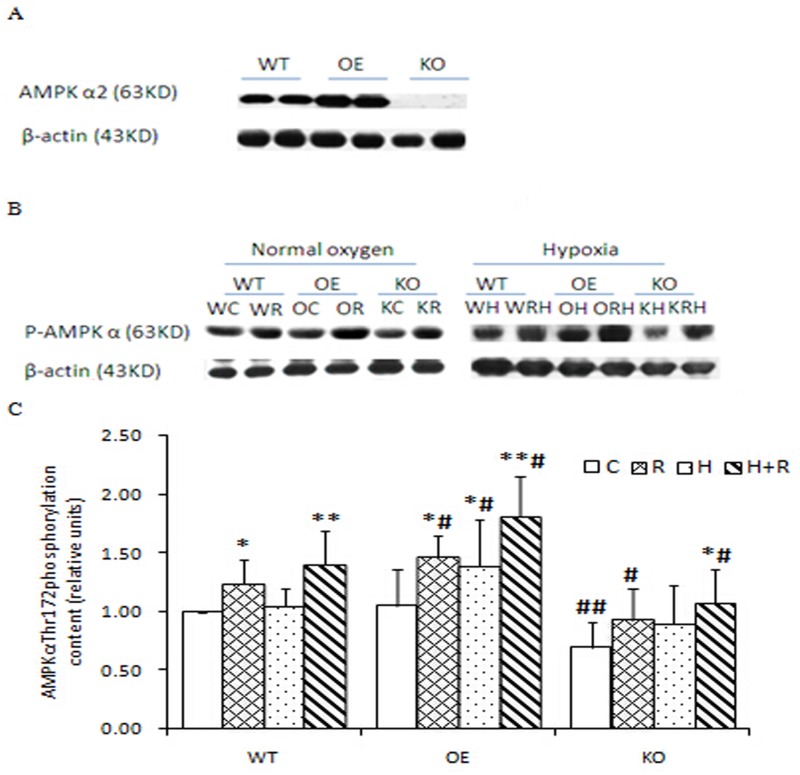
AMPKα2 protein content (**Section A**) in muscle of wild-type (WT), over expression (OE), knockout (KO) mice and AMPKα Thr^172^ phosphorylation (**Section B**) in the control (C), running (R), hypoxia (H), and running + hypoxia (R+H) groups of the three geno-type mice. Data are presented as mean±SD (**Section C**). * significantly different from the control group, p<0.05; ** p<0.01. # significantly different from the same group in WT, p<0.05; ## p<0.01. WC = WT control, WR = WT running, OC = OE control, OR = OE running, KC = KO control, KR = KO running, WH = WT hypoxia, WRH = WT running + hypoxia, OH = OE hypoxia, ORH = OE running + hypoxia, KH = KO hypoxia, and KRH = KO running + hypoxia.

AMPKα2 was over expressed and knocked out in the OE and KO mice, respectively. The effects of gene modification were proved by AMPKα2 protein levels in the skeletal muscle (Fig 1A). The protein was detected with the antibody to AMPK2α in WT mice, was significantly elevated in OE mice, but without detectable signal in KO mice.

As an indicator of AMPK activation, phosphor-AMPKα (p-AMPKα) (Thr172) was measured in the muscle samples by Western blot, using phosphor-specific antibody. The antibody is able to detect both α1 and α2 isoforms of AMPK, but cannot detect the β or γ subunits of AMPK.

In WT mice, AMPK activation was significantly increased in skeletal muscle by treadmill running as indicated by 24% (p<0.05) elevation over the control in phosphor-AMPK signal (Fig 1B and 1C). The AMPK activation was further enhanced by running in hypoxia for 41% (p<0.01) increase in the phosphorylation signal. In OE mice, the basal phosphorylation signal was same to that of WT mice (Fig 1B and 1C). However, OE mice showed 23% (p<0.05) and 40% (p<0.05) more AMPK phosphorylation than WT mice in the running and hypoxic running conditions, respectively. In KO mice, the protein of AMPKα2 was not detectable due to gene knockout (Fig 1A), but the phosphorylation of AMPKα (AMPKα1/α2) was detected (Fig 1B). Phosphor-AMPKα was induced by running and hypoxic running (Fig 1B and 1C). However, the increases were 30% (p<0.01) and 34% (p<0.05) lower than those of WT mice in the same conditions, respectively (Fig 1B and 1C).

Hypoxia alone did not change AMPK activation in WT mice, although it was a factor that induced AMPK activation in OE mice. AMPK activation was enhanced following four weeks of running training and the enhancement was stronger after the running plus hypoxia treatment. The AMPK responses in the R+H groups were up-regulated in OE mice (p<0.01), but down-regulated in KO mice (p<0.05) of AMPKa2, compared to the R+H group of WT mice (Fig 1B and 1C).

### The changes of nuclear PPARα protein expression in muscle lysates

**Fig 2:**
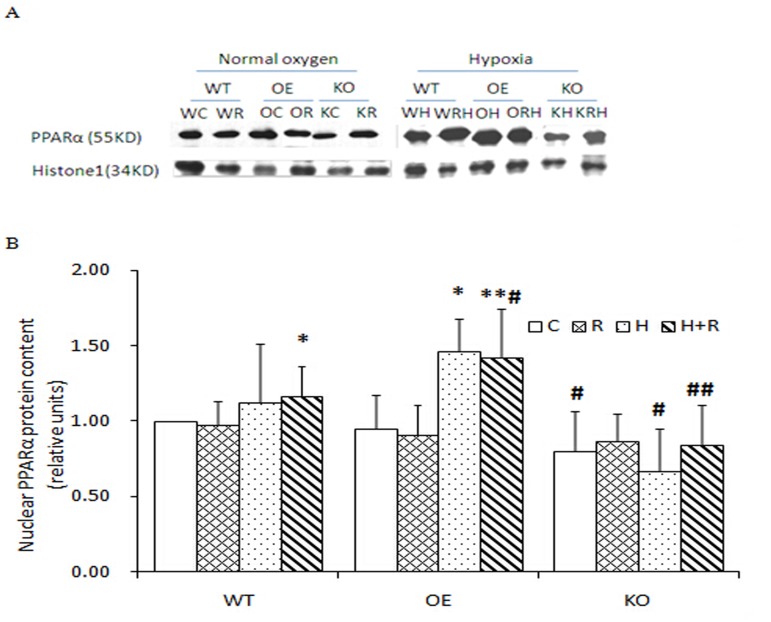
Nuclear PPARα protein content (**Section A**) in muscle of wild-type (WT), over expression (OE), knockout (KO) mice of the control (C), running (R), hypoxia (H), and running + hypoxia (R+H) groups. Data are presented as mean±SD (**Section B**). * significantly different from the control group, p<0.05; ** p<0.01. # significantly different from the same group in WT, p<0.05; ## p<0.01. WC = WT control, WR = WT running, OC = OE control, OR = OE running, KC = KO control, KR = KO running, WH = WT hypoxia, WRH = WT running + hypoxia, OH = OE hypoxia, ORH = OE running + hypoxia, KH = KO hypoxia, and KRH = KO running + hypoxia.

In normoxia, the nuclear PPARα protein was not significantly changed by running exercise in WT mice (Fig 2, WT). The same result was observed in OE and KO mice (Fig 2A, OE and KO). In hypoxia, the protein was increased by the treadmill running for 17% (p<0.05) in WT mice (Fig 2B, WT). An increase (50%, P<0.05) was observed in OE mice in the hypoxia condition, but the increase was not further changed by the running plus hypoxia treatment (Fig 2B, OE). The protein expressions were significantly lower in C and R+H groups of KO mice compared with those in WT (Fig 2B, KO). These results suggest that the PPARα protein may be increased in muscle by four weeks of hypoxia training. The increase is dependent on AMPK activation.

### The change of MCAD mRNA level in muscle lysates

**Fig 3:**
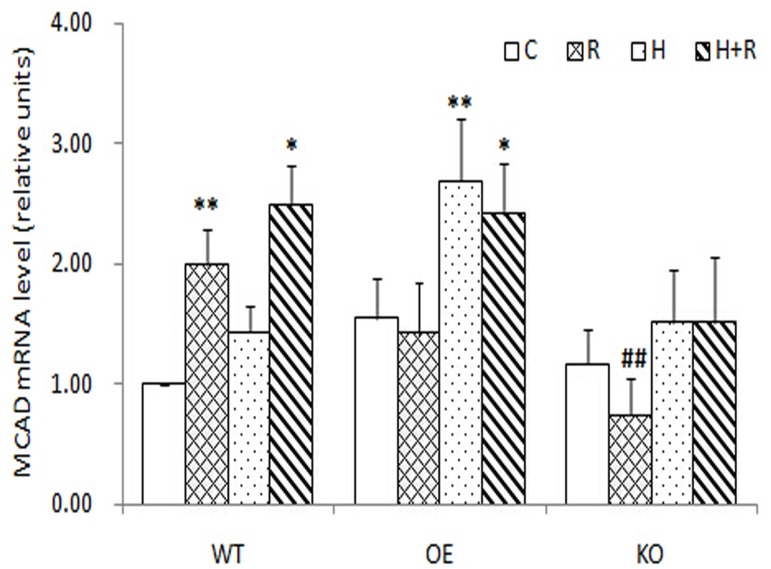
MCAD mRNA in C, R, H, R+H groups of the three geno-type mice. * significantly different from the control group, p<0.05; ** p<0.01. # significantly different from the same group in WT, p<0.01; ## p<0.01.

The MCAD mRNA expression in the skeletal muscle was significantly increased by 2 and 2.39 times after four weeks of R or R+H intervention in WT mice, respectively, but hypoxia alone did not change MCAD mRNA level significantly. After the R+H intervention, OE mice had a similar level of MCAD mRNA expression but KO mice showed a lower level, compared with that of WT. These results suggest that MCAD mRNA expression after intermittent hypoxic training may be controlled via an AMPKα2-dependent manner (Fig 3).

## Discussion

PPAR and AMPK have been the subject of intense investigations in molecular biology, physiology, and pharmacology since they are critical regulators of fatty acid oxidation [29–31] and may be involved to modulate metabolic risk factors, such as dyslipidemia, obesity, and type 2 diabetes [6,32,33]. From the perspective of metabolic regulation, AMPK responds to intracellular ATP depletion, while PPARα induces the expression of genes coding for enzymes and proteins involved in increasing cellular ATP yields [34]. It is logical to deduce that there would be a relationship between AMPK and PPARα. However, there was a paucity of research relating to the possible relationship *in vivo*. Therefore, the PPARα expression in skeletal muscles from AMPKα2 OE, AMPKα2 KO, and corresponding WT mice after intermittent hypoxic training were examined for the first time in the present study.

Four weeks of exercise training or intermittent hypoxic treatment alone did not make any change in the nuclear PPARα protein content. This result was supported by the short-term (shorter than four weeks) exercise studies in human skeletal muscle [35,36], while it was different to the long-term exercise studies, in which 6–12 weeks of exercise training achieved significant increases in PPARα protein or PPARα mRNA in human skeletal muscle [37,38] or in rat cardiac muscle [13]. These results suggested that the change in PPARα protein expression in muscle tissues might need a longer duration. Different to the separate effects of exercise or hypoxia alone, however, the four-week combined R+H training increased PPARα nuclear protein content and its target gene MCAD mRNA level in skeletal muscles of WT mice significantly. This outcome supports the hypothesis of the present study.

It has been well documented that the higher expression of PPARα had been thought to stimulate lipid metabolism in tissues [39] and the PPARα target gene MCAD is one of four different chain-length-specific enzymes that catalyze the initial reaction in the mitochondrial fatty acid β-oxidation cycle [40]. Substrates for MCAD include medium-chain length (C6–C12) acyl-CoA thioesters derived from (i) medium-chain fatty acids that enter mitochondria by diffusion, (ii) products of mitochondrial β-oxidation of saturated and unsaturated long-chain fatty acids, and (iii) products of peroxisomal β-oxidation of long-chain and very long-chain fatty acids. Because these diverse pathways of fatty acid oxidation converge at this point, MCAD catalyzes a pivotal step in cellular fatty acid metabolism. Thus, it seemed reasonable to think that the intermittent hypoxic training could be used as a potential treatment for fat loss. However, future studies are needed to test this possibility.

When skeletal muscles face physiological challenges (e.g., exercise and hypoxia), AMPK becomes activated in response to changes in cellular energy status and serves to inhibit ATP-consuming pathways and activate pathways involved in carbohydrate and fatty acid metabolism to restore ATP levels [17]. It has been reported that the AMPKα2 was much more abundant than the AMPKα1 in skeletal muscles [41] and the actions of AMPK in skeletal muscle tissues were mediated mainly through the AMPKα2 isoform [25], even though AMPK was also activated via the phosphorylation of its α1 subunit. To explore whether AMPKα2 were involved in the regulation of PPARα after the four-week intermittent hypoxic training, we measured PPARα protein, its target gene MCAD mRNA, and p-AMPKα. As the results, the intermittent hypoxic training achieved a much significant increase in AMPK activation in WT muscles than that of the exercise training or intermittent hypoxia alone. This observation is in agreement with previous studies, in which the p-AMPKα content in the gastrocnemius muscle was increased by 2.5 times after six weeks of swimming plus hypoxia intervention in lean Zucker rats, but swim training and hypoxia alone did not change the p-AMPKα content [42]. Moreover, compared the changes in p-AMPKα among three genotypes of mice following the intermittent hypoxic training, we found that the OE mice showed a higher value, while the KO mice a lower value than that of the WT mice. Therefore, the intermittent hypoxic training induced an increase in AMPK activation significantly affected by overexpression and knockout of the AMPKα2-isoform. Meanwhile, our findings indicated that overexpression of the α2-isoform was associated with 25% higher and knockout of the α2-isoform was associated with 33% lower PPARα protein content in muscles of the R+H group, compared with that of WT. Likewise, the changes of MCAD mRNA level in skeletal muscles of the R+H group followed a similar trend as that of PPARα protein content in OE or KO mice, respectively. In summary, it is reasonable to deduce the hypoxic training approach elicited a profound increase in AMPK activation and induced PPARα nuclear protein expression, which occurred via an AMPKα2 isoform dependent manner. This result supports the hypothesis of our study.

Previous studies [6,21], but not all [20,22,23], agreed with our observation about AMPK activation impacting positively on PPARα. Some studies demonstrated that muscle AMPK activation by AICAR induced mRNA expression of PPARα target genes [6,21]. Conversely, other studies have also provided evidence that activated AMPK by AICAR robustly inhibited PPARα transcriptional activity [20,22,23]. In addition, it has been reported that AMPK, independently of AMPK activity, activated PPARα by directly binding of AMPKα to the PPARα ligand-binding domain [20]. Collectively, different tissues and study designs might lead to this conflicting situation. The existing investigations have mostly carried out by cultured cells *in vitro* and used AICAR as the AMPK activator. And yet, little information on the relationship of AMPK and PPARα *in vivo* has been obtained from animal or human studies in which intermittent hypoxic training was employed as the AMPK activator. Indeed, our present study has indicated that PPARα protein expression in mouse skeletal muscle was increased by four weeks of intermittent hypoxic training and the increase was associated with AMPK activation occurred via an AMPKα2 isoform dependent manner, but the mechanisms were not examined in this study. Further studies are needed to elucidate the molecule signaling role in this change.

It is important to acknowledge the limitations of our study, we did not assess PPARδ, which is also abundant in skeletal muscle. Similar to PPARα, PPARδ regulates fatty acid catabolism and shares the common target gene of MCAD with PPARα [43]. Exercise, in both acute and prolonged forms, increased the expression of PPARδ in skeletal muscle [44,45]. In addition, we did not investigate the mRNA changes of other PPARα-regulated target genes except for MCAD. Therefore, it would be desirable to assess PPARδ expression in parallel with PPARα and the mRNA changes of other PPAR α-regulated target genes in future studies. New evidence from the additional experiments would give more comprehensive insights into the intermittent hypoxic training-induced benefits on fatty acid catabolism.

In conclusion, the major findings of this study were that four weeks of intermittent hypoxic training significantly increased nuclear PPARα protein and the increase was associated with AMPK activation via an AMPKα2 isoform dependent manner. More studies are needed to deeply understand the possible effects of this training model on promoting fatty acid oxidation.
